# Flap perfusion monitoring with an attached surface probe in microvascular reconstruction of the oral cavity

**DOI:** 10.1007/s00784-023-05177-x

**Published:** 2023-07-31

**Authors:** Mark Ooms, Philipp Winnand, Marius Heitzer, Florian Peters, Anna Bock, Marie Katz, Frank Hölzle, Ali Modabber

**Affiliations:** Department of Oral and Maxillofacial Surgery, University Hospital RWTH Aachen, Pauwelsstraße 30, 52074 Aachen, Germany

**Keywords:** Microvascular head and neck reconstruction, Free flap, Flap perfusion monitoring, Oxygen-2-see analysis system, Attached surface probe

## Abstract

**Objectives:**

Postoperative flap monitoring is essential in oral microvascular reconstruction for timely detection of vascular compromise. This study investigated the use of attached surface probes for the oxygen-2-see (O2C) analysis system (LEA Medizintechnik, Germany) for intraoral flap perfusion monitoring.

**Materials and methods:**

The study included 30 patients who underwent oral reconstruction with a microvascular radial-free forearm flap (RFFF) or anterolateral thigh flap (ALTF) between 2020 and 2022. Flap perfusion was measured with attached (3-mm measurement depth) and unattached surface probes (2- and 8-mm measurement depths) for the O2C analysis system at 0, 12, 24, 36, and 48 h postoperatively. Flap perfusion monitoring with attached surface probes was evaluated for cut-off values for flap blood flow, hemoglobin concentration, and hemoglobin oxygen saturation indicative of vascular compromise and for accuracy and concordance with unattached surface probes.

**Results:**

Three RFFFs were successfully revised, and one ALTF was unsuccessfully revised. The cut-off values indicative of vascular compromise for flap perfusion monitoring with attached surface probes were for RFFF and ALTF: blood flow < 60 arbitrary units (AU) and < 40AU, hemoglobin concentration > 100AU and > 80AU (both > 10% increase), and hemoglobin oxygen saturation < 40% and < 30%. Flap perfusion monitoring with attached surface probes yielded a 97.1% accuracy and a Cohen’s kappa of 0.653 (*p* < 0.001).

**Conclusions:**

Flap perfusion monitoring with attached surface probes for the O2C analysis system detected vascular compromise accurately and concordantly with unattached surface probes.

**Clinical relevance:**

Attached surface probes for the O2C analysis system are a feasible option for intraoral flap perfusion monitoring.

## Introduction

Microvascular reconstruction of the head and neck region with free flaps offers functional and esthetic outcomes as well as high overall success rates [1, 2]. However, the use of microvascular free flaps remains challenging due to transient flap failure with the need for flap revision and terminal flap failure with loss of the flap, which is associated with high patient burden and the need for additional surgeries or lasting functional and esthetic compromise [1, 3, 4].

Microvascular free flap perfusion, as a prerequisite for flap viability, initially depends entirely on continuous arterial inflow and venous outflow through a patent microvascular anastomosis, and ischemia of the flap related to vascular compromise is associated with deleterious effects such as microthrombi formation, ultimately leading to irreversible microcirculation failure and subsequently flap failure [5–9]. Given the inverse relationship of the time interval between the onset of vascular compromise and intervention and the flap salvage rate, postoperative flap monitoring for timely detection of vascular compromise is crucial [8, 10, 11]. In this context, the most commonly performed method of clinical monitoring is based on flap color, surface temperature, capillary refill, and pricking tests; however, such monitoring is limited by several constraints, such as the need for clinical experience, lack of objective values, and delay between the onset of vascular compromise and the appearance of clinical changes [8, 12, 13].

Therefore, the oxygen-2-see (O2C) analysis system (LEA Medizintechnik, Germany) was developed as an objective method for flap perfusion monitoring, and cut-off values indicative of vascular compromise have been established for unattached surface probes measuring flap perfusion at tissue depths of 2 mm and 8 mm [7, 9]. However, attached surface probes measuring flap perfusion at a tissue depth of 3 mm to monitor flap perfusion with the O2C analysis system have potential advantages, such as constant probe pressure and consistent location of the measurement area, both of which may affect the measurements; moreover, attached surface probes have the potential to provide the basis for continuous monitoring [14–16].

This study aimed to evaluate the use of attached surface probes for monitoring flap perfusion with the O2C analysis system in microvascular head and neck reconstruction for the detection of vascular compromise.

## Materials and methods

### Study population

This study was approved by the local ethics committee of the Medical Faculty RWTH Aachen University (EK 22–358). All study data were prospectively collected and retrospectively analyzed.

The study population consisted of 30 patients who had undergone reconstruction with a microvascular free flap — i.e., a radial free forearm flap (RFFF) or an anterolateral thigh flap (ALTF) — in the oral cavity between 2020 and 2022 in our Department of Oral and Maxillofacial Surgery after ablative surgery for malignant or nonmalignant disease. Patients with complete data records who were older than 18 years were included.

Data were obtained from clinical records and O2C analysis system measurement records. Surgery duration and flap ischemia duration were calculated as the time interval between the first incision and the last suture or between the separation of the flap pedicle vessel at the donor site and the release of the vascular clamp to initiate flap perfusion at the recipient side after anastomosis. Flap revision was defined in terms of flaps whose anastomosis was surgically revised with a return to the operating room, and flap failure was defined in terms of flaps that were completely lost due to tissue necrosis and subsequently removed.

All surgical procedures were performed under general anesthesia, and all patients were monitored postoperatively in the intensive care unit. All patients received invasive mechanical ventilation and analgosedation at least until the next morning.

### Flap perfusion monitoring

**Fig. 1:**
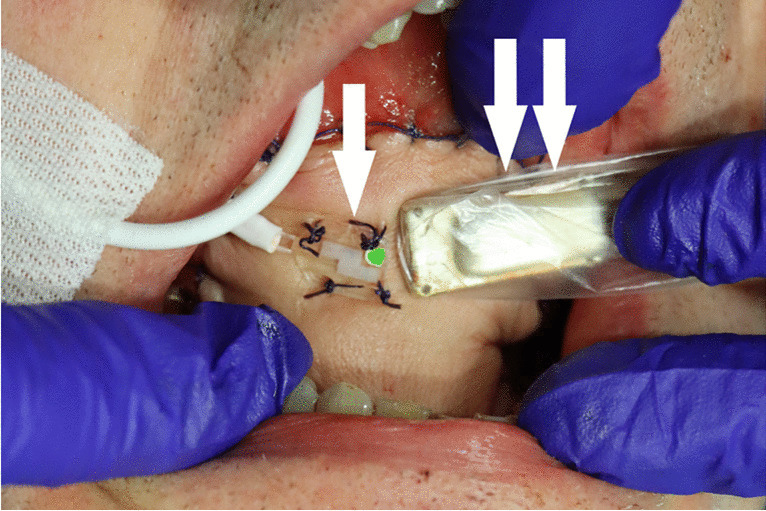
Flap perfusion monitoring setting. Measurement of perfusion of a RFFF localized in the mouth floor right in 3-mm tissue depth with an attached surface probe (single arrow) and in 2-mm and 8-mm tissue depth with an unattached surface probe (double arrows)

Flap perfusion was simultaneously monitored with the O2C analysis system (oxygen-to-see (O2C), LEA Medizintechnik, Giesen, Germany) with an unattached surface probe (type LFX90) as well as with an attached surface probe (type LF2) at 0, 12, 24, 36, and 48 h postoperatively (Fig. 1). The attached surface probe, measuring perfusion at a tissue depth of 3 mm, was fixed with four sutures in the middle of the RFFF and at a distance of 1 cm from the perforator vessel, approximately in the middle of the ALTF. The unattached probe, measuring perfusion at tissue depths of 2 mm and 8 mm, was held in the same position approximately parallel to the attached surface probe for each measurement.

The O2C analysis system for measuring flap tissue perfusion is based on the following two principles: laser light Doppler spectroscopy (830 nm; 30 mW) for the determination of flap blood flow (arbitrary units [AU]) and white light spectroscopy (500–800 nm; 50 W) for the determination of hemoglobin concentration (AU) and hemoglobin oxygen saturation (%) [7, 17]. The probe transmits the laser and white light into the flap tissue and then detects the backscattered light [7, 17]. Blood flow values are calculated as the product of erythrocyte quantity, based on the analysis of the sum of light absorbances, and velocity, based on the analysis of the Doppler shift of light wave frequencies due to the movement of the erythrocytes in the blood vessels [7, 17]. Hemoglobin concentration and hemoglobin oxygen saturation values are calculated as the sum of light absorbances, and the color change of light absorbances in comparison to prerecorded hemoglobin spectra with defined oxygen saturation, respectively [7, 17]. For both surface probes, the measurement time interval was 10 s.

### Statistical analysis

Clinical data are expressed as numbers (with percentages) or medians (with interquartile ranges). Measurement data for flap perfusion — i.e., flap blood flow, hemoglobin concentration, and hemoglobin oxygen saturation — obtained with the unattached surface probe were used as a reference standard, with the following cut-off values indicative of vascular compromise according to previous studies: blood flow at 8-mm tissue depth: RFFF < 20AU and ALTF < 15AU; hemoglobin concentration at 8-mm tissue depth: RFFF and ALTF both > 30% increase; hemoglobin oxygen saturation at 8-mm tissue depth: RFFF < 15% and ALTF < 10%; blood flow at 2-mm tissue depth: RFFF < 10AU and ALTF < 5AU; hemoglobin concentration at 2-mm tissue depth: RFFF and ALTF both > 30% increase; and hemoglobin oxygen saturation at 2-mm tissue depth: RFFF < 15% and ALTF < 10% [7, 9]. Cut-off values indicative of vascular compromise for flap perfusion monitoring with attached surface probes were derived based on flap perfusion measurement values from not revised and revised flaps separately for RFFF and ALTF. Sensitivity, specificity, and accuracy for flap perfusion monitoring with attached surface probes and Cohen’s kappa for determining concordance between flap perfusion monitoring with attached surface probes and flap perfusion monitoring with unattached surface probes were calculated according to commonly used definitions [18, 19]. Values of *p* < 0.05 were considered to be statistically significant. The statistical analysis was performed using SPSS version 28 (SPSS, IBM, New York, USA).

## Results

### Study population

The study population was composed of 30 patients, 11 men and 19 women (Table 1). Twenty-seven patients underwent reconstruction with a RFFF, and three patients underwent reconstruction with an ALTF. A total of four flaps were revised (3 RFFF primarily due to venous vascular compromise and 1 ALTF primarily due to arterial vascular compromise), and one flap was lost (ALTF).

**Table 1:** Characteristics of the study population

Number	30
Sex (n)	
Male	11 (36.7%)
Female	19 (63.3%)
Age (years)	66.0 (12.0)
BMI (kg/m^2^)	25.5 (10.0)
ASA (n)	
1	0 (0.0%)
2	11 (36.7%)
3	19 (63.3%)
4	0 (0.0%)
Flap type (n)	
RFFF	27 (90.0%)
ALTF	3 (10.0%)
Flap location (n)	
Tongue	9 (30.0%)
Mouth floor	6 (20.0%)
Mandibular	8 (26.7%)
Maxilla	4 (13.3%)
Cheek	3 (10.0%)
Arterial anastomosis recipient vessel (n)	
Facial artery	24 (80.0%)
Lingual artery	2 (6.7%)
Superior thyroid artery	4 (13.3%)
Venous anastomosis recipient vessel (n)	
Internal jugular vein	19 (63.3%)
Internal jugular vein + another vein	7 (23.3%)
Another vein	4 (13.3%)
Surgery duration (min)	510.5 (173.0)
Flap ischemia duration (min)	83.0 (33.0)
Flap revision (n)	
No	26 (86.7%)
Yes	4 (13.3%)
Flap survival (n)	
No	1 (3.3%)
Yes	29 (96.7%)

Parameters are indicated as numbers (with percentage) for categorical data (sex, ASA, flap type, flap location, arterial anastomosis recipient vessel, venous anastomosis recipient vessel, flap revision, flap survival) or median (with interquartile range) for metric data (age, BMI, surgery duration, flap ischemia duration); other veins = facial vein, superior thyroid vein

*BMI* body mass index, *ASA* American Society of Anesthesiologists score, *RFFF* radial free forearm flap, *ALTF* anterolateral thigh flap

### Flap perfusion measurement values

**Fig. 2:**
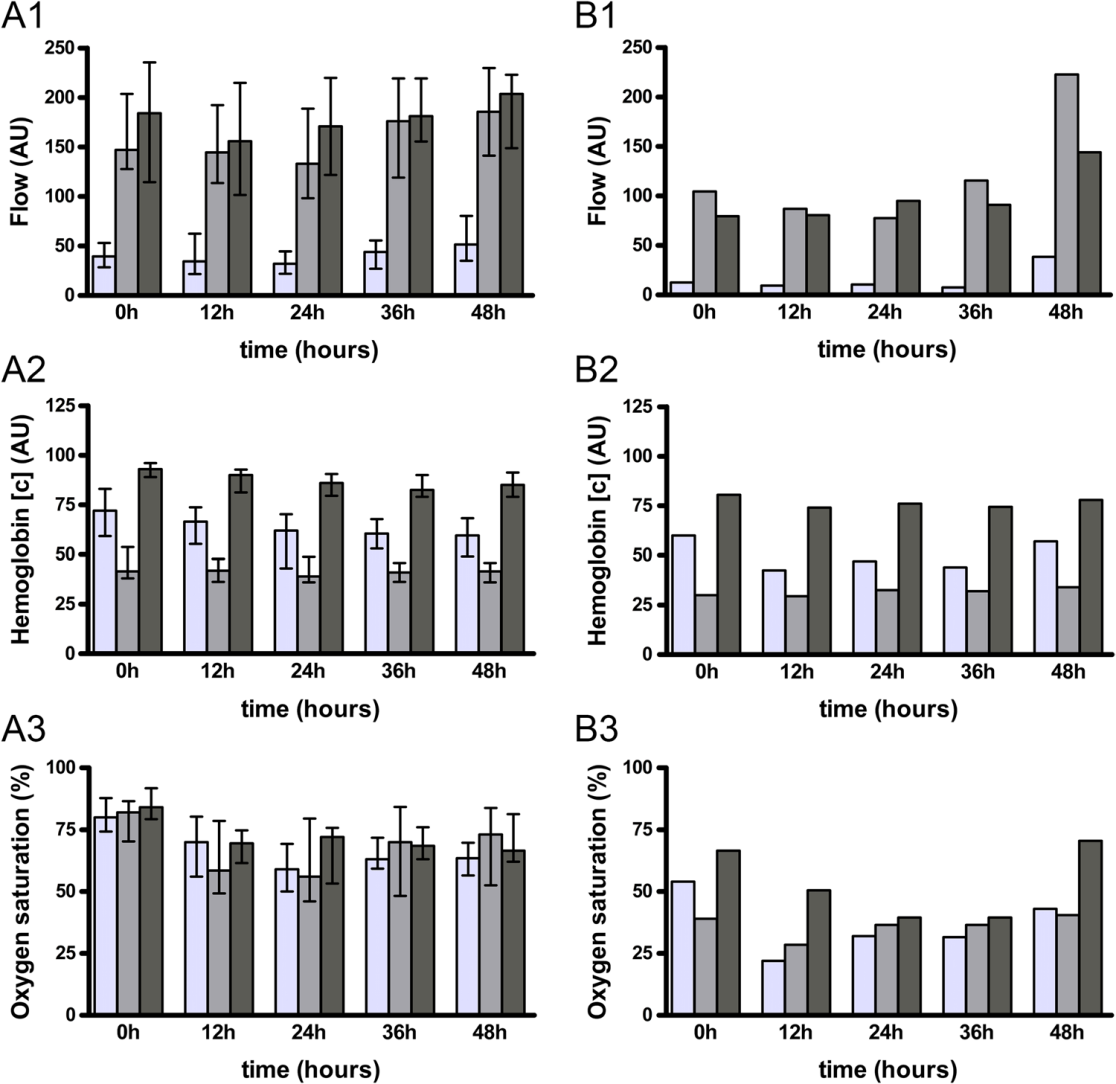
Flap perfusion monitoring of not revised flaps. Data shown as median (with (**A**) and without (**B**) interquartile range) separately for RFFF (**A**) and ALTF (**B**) for blood flow (1), hemoglobin concentration (2), and hemoglobin oxygen saturation (3) in 2-mm (light grey), 3-mm (middle grey), and 8-mm (dark grey) tissue depths at different measurement timepoints (0 h postoperatively, 12 h postoperatively, 24 h postoperatively, 36 h postoperatively, 48 h postoperatively); *AU* arbitrary units

**Fig. 3:**
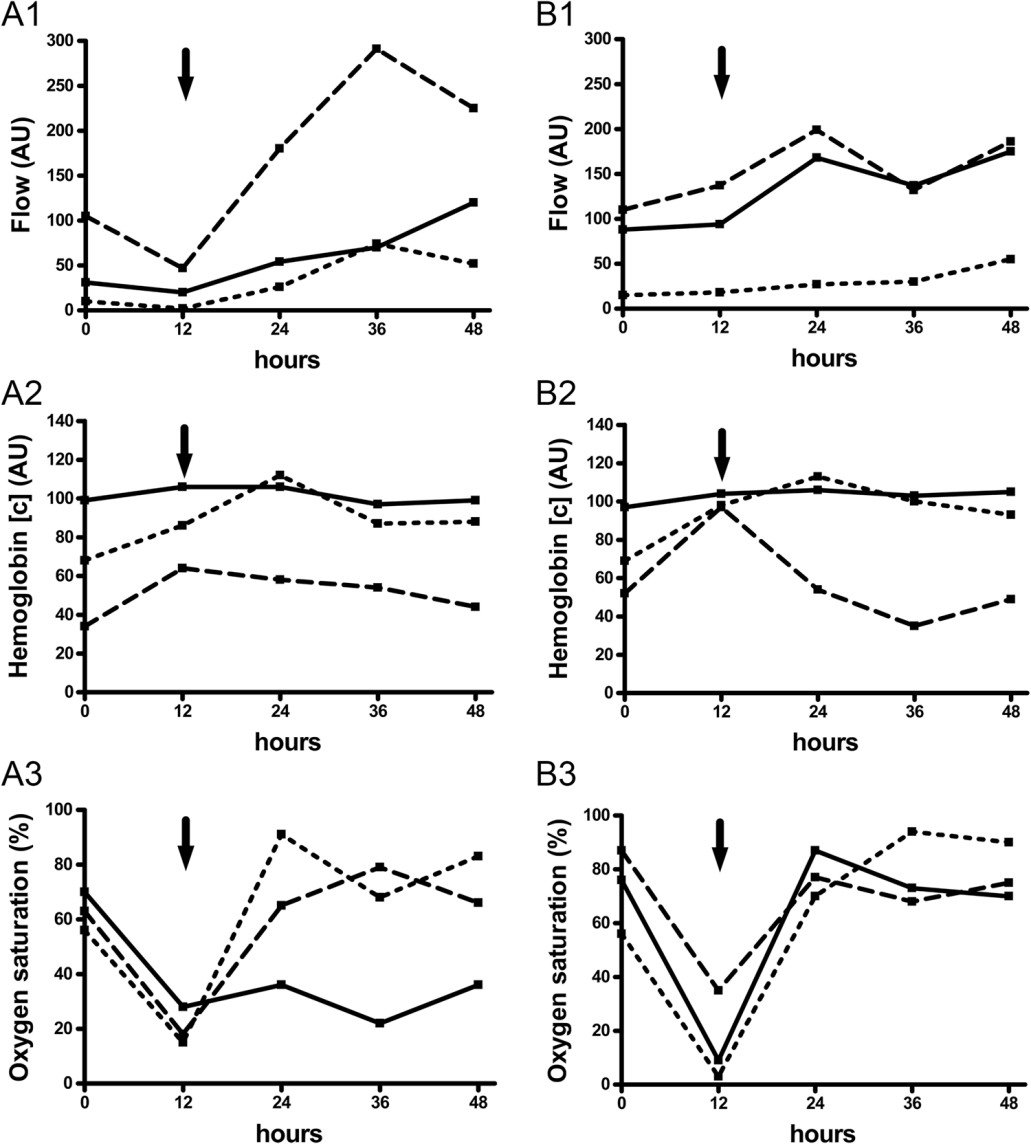

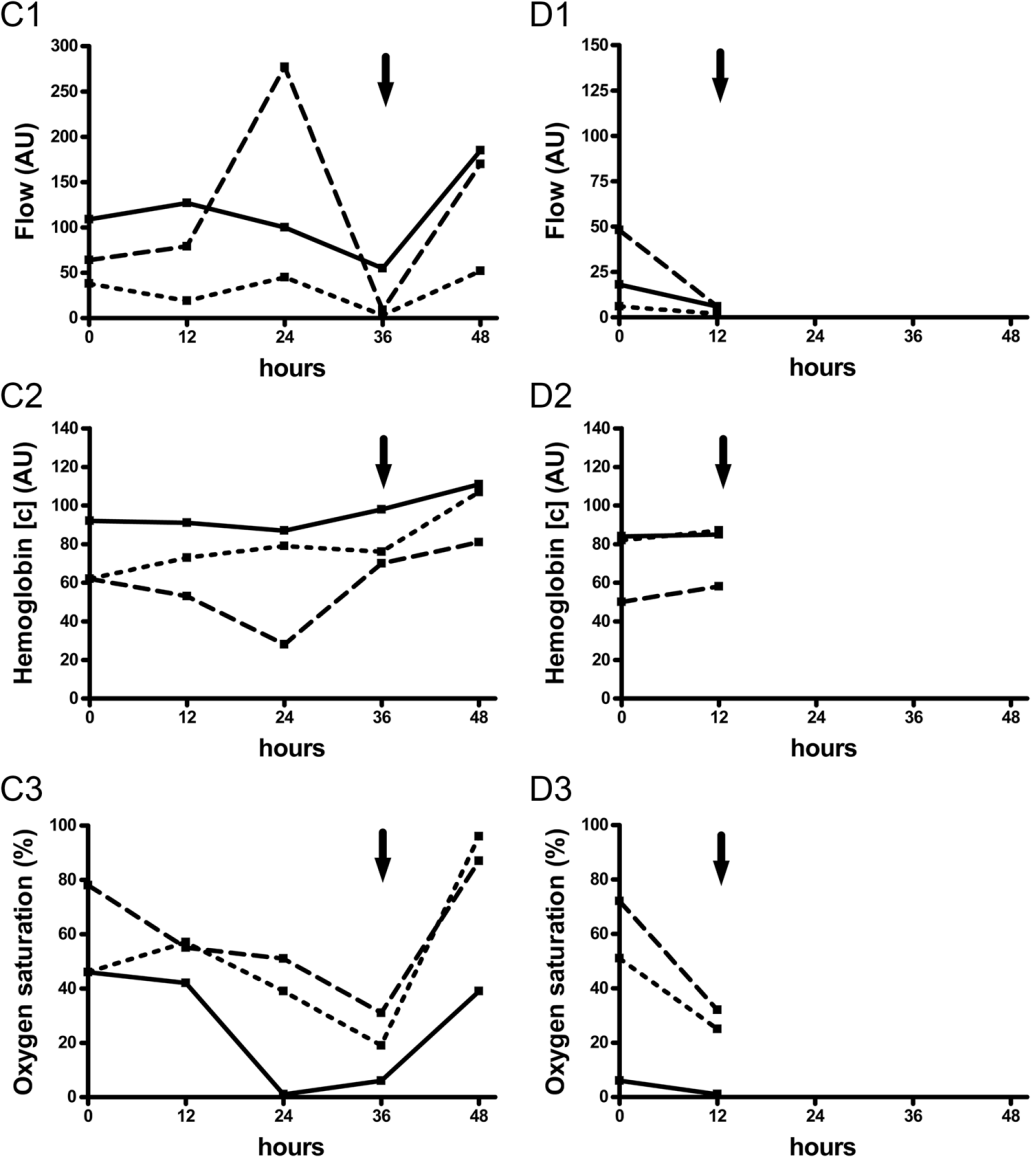
Flap perfusion monitoring of revised flaps. Data shown as individual perfusion values for three revised RFFF (**A**, **B**, **C**) primarily due to venous vascular compromise and one revised ALTF (**D**) primarily due to arterial vascular compromise separately for blood flow (1), hemoglobin concentration (2), and hemoglobin oxygen saturation (3) in 2-mm (dotted line), 3-mm (straight line), and 8-mm (dashed line) tissue depths at different measurement timepoints (0 h postoperatively, 12 h postoperatively, 24 h postoperatively, 36 h postoperatively, 48 h postoperatively); arrow: flap perfusion measurement before flap revision (ALTF revision was not successful); [c] concentration, AU arbitrary units

Flap perfusion measurement values for blood flow, hemoglobin oxygenation, and hemoglobin oxygen saturation for not revised flaps are described and presented separately for RFFF and ALTF at 0, 12, 24, 36, and 48 h postoperatively (Table 2; Fig. 2). In addition, flap perfusion measurement values for blood flow, hemoglobin oxygenation, and hemoglobin oxygen saturation for revised flaps are presented separately for each flap at 0, 12, 24, 36, and 48 h postoperatively (Fig. 3).

**Table 2:** Measurement values for flap perfusion monitoring of not revised flaps

Tissue depth	Timepoint of measurement				
	0 h	12 h	24 h	36 h	48 h
RFFF (n = 24)					
Blood flow (AU)					
2 mm	39.5 (25.0)	34.5 (41.0)	32.0 (23.0)	44.0 (29.0)	51.5 (45.0)
8 mm	147.0 (76.0)	144.5 (79.0)	133.0 (91.0)	176.0 (100.0)	185.5 (89.0)
3 mm	184.0 (121.0)	156.0 (113.0)	171.0 (98.0)	181.0 (64.0)	203.5 (74.0)
Hemoglobin concentration (AU)					
2 mm	72.0 (24.0)	66.5 (19.0)	62.0 (27.0)	60.5 (15.0)	59.5 (19.0)
8 mm	41.5 (16.0)	42.0 (12.0)	39.0 (13.0)	41.0 (10.0)	41.5 (10.0)
3 mm	93.0 (7.0)	90.0 (12.0)	86.0 (11.0)	82.5 (11.0)	85.0 (12.0)
Hemoglobin oxygen saturation (%)					
2 mm	80.0 (14.0)	70.0 (24.0)	59.0 (19.0)	63.0 (13.0)	63.5 (13.0)
8 mm	82.0 (16.0)	58.5 (29.0)	56.0 (34.0)	70.0 (36.0)	73.0 (31.0)
3 mm	84.0 (13.0)	69.5 (13.0)	72.0 (23.0)	68.5 (13.0)	66.5 (19.0)
ALTF (n = 2)					
Blood flow (AU)					
2 mm	12.5 (–)	9.5 (–)	10.5 (–)	7.5 (–)	38.5 (–)
8 mm	104.5 (–)	87.0 (–)	77.5 (–)	115.5 (–)	222.5 (–)
3 mm	79.5 (–)	80.5 (–)	95.0 (–)	91.0 (–)	144.0 (–)
Hemoglobin concentration (AU)					
2 mm	60.0 (–)	42.5 (–)	47.0 (–)	44.0 (–)	57.0 (–)
8 mm	30.0 (–)	29.5 (–)	32.5 (–)	32.0 (–)	34.0 (–)
3 mm	80.5 (–)	74.0 (–)	76.0 (–)	74.5 (–)	78.0 (–)
Hemoglobin oxygen saturation (%)					
2 mm	54.0 (–)	22.0 (–)	32.0 (–)	31.5 (–)	43.0 (–)
8 mm	39.0 (–)	28.5 (–)	36.5 (–)	36.5 (–)	40.5 (–)
3 mm	66.5 (–)	50.5 (–)	39.5 (–)	39.5 (–)	70.5 (–)

Perfusion measurement values are indicated as median (with interquartile range) for each measurement time point (0 h postoperatively, 12 h postoperatively, 24 h postoperatively, 36 h postoperatively, 48 h postoperatively) separately for 2-mm, 8-mm and 3-mm tissue depth and RFFF and ALTF for not revised flaps

*AU* arbitrary units

### Determination of cut-off values

Based on the perfusion measurement values for not revised and revised flaps, the following cut-off values indicative of vascular compromise for flap perfusion monitoring with attached surface probes were derived: blood flow RFFF < 60AU and ALTF < 40AU; hemoglobin concentration RFFF > 100AU or > 10% increase and ALTF > 80AU or > 10% increase; and hemoglobin oxygen saturation RFFF < 40% and ALTF < 30% (Table 3).

**Table 3:** Cut-off values for flap perfusion monitoring

Flap type	Cut-off values		
	Blood flow	Hemoglobin [c]	Hemoglobin oxygen saturation
RFFF	< 60 AU	> 100 AU/ > 10% increase	< 40%
ALTF	< 40 AU	> 80 AU/ > 10% increase	< 30%

Cut-off values for blood flow, hemoglobin concentration and hemoglobin oxygen saturation indicating vascular compromise for flap perfusion monitoring with attached surface probes; abbreviations

*AU* arbitrary units, *[c]* concentration

### Evaluation of sensitivity, specificity, accuracy, and concordance

Based on the derived cut-off values indicative of vascular compromise for flap perfusion monitoring with attached surface probes, the following values for sensitivity, specificity, accuracy, and concordance in detecting vascular compromise were obtained: sensitivity, 100.0%; specificity, 97.1%; accuracy, 97.1%; and Cohen’s kappa, 0.653 (*p* < 0.001).

## Discussion

This study evaluated postoperative flap perfusion monitoring with attached surface probes for the O2C analysis system in terms of determining cut-off values indicative of vascular compromise. Based on these cut-off values, sensitivity, specificity, accuracy, and concordance with flap perfusion monitoring using standard unattached surface probes were assessed in terms of detecting vascular compromise [7, 9].

Postoperative flap monitoring is considered crucial in microvascular free flap reconstruction of the head and neck region, as vascular compromise occurs, and timely detection and intervention are prerequisite for flap salvage [8, 10, 11]. In this context, the O2C analysis system was developed as a reliable and objective method for flap monitoring based on flap perfusion measurement, as flap perfusion is a prerequisite for flap viability [7–9, 20, 21]. Nonetheless, flap perfusion can be assessed with several other methods, including indocyanine green angiography with the disadvantages of invasiveness and the need for intravenous administration of agents, pin prick test with the disadvantages of invasiveness and subjective interpretation, and basic doppler assessment of vascular flow with the disadvantages of subjective interpretation and difficulty in distinguishing between arterial and venous compromise [7–9]. The O2C analysis system is standardly used with unattached surface probes that measure flap perfusion at tissue depths of 2 mm and 8 mm, and the attendant cut-off values indicative of vascular compromise have been previously evaluated and approved [7, 9]. However, attached surface probes that measure flap perfusion only at one tissue depth of 3 mm, but are smaller and lighter, could offer potential advantages for flap perfusion measurement, such as consistent probe pressure and location of the measurement area over the entire postoperative monitoring course — both of which are likely to influence flap perfusion measurement — and could provide the basis for continuous flap perfusion monitoring [14–16].

In this study, cut-off values indicative of vascular compromise were determined for flap perfusion monitoring with attached surface probes for the O2C analysis system. Cut-off values indicative of vascular compromise differed between the attached and unattached surface probes, reflecting differences in measurement depth — i.e., 2 mm and 8 mm and 3 mm, respectively — and thus differences in the skin microvasculature depending on the skin layer examined — i.e., the dermis containing the superficial papillary and the deeper reticular plexus and the subcutaneous tissue containing the subcutaneous plexus [22, 23]. Differences in vessel density and diameter between these plexus formations, for example, are likely to affect blood flow and hemoglobin concentration in terms of the relationship between vessel diameter, resistance, and blood flow [22–24]. In addition, hemoglobin oxygen saturation has been shown to be dependent on blood flow [7]. When considering the differences in perfusion measurements in this study, it should be noted that in addition to the measurement depth, the measurement area was not identical between the two surface probes [14, 15]. Furthermore, the influence of the intraoral moist environment on the perfusion measurement, which is likely to be more pronounced with attached probes since they cannot be removed for drying, could not be excluded [25].

Notably, in line with cut-off values indicative of vascular compromise for unattached probes for flap perfusion monitoring with the O2C analysis system determined in previous studies and used as the reference standards in this study, cut-off values indicative of vascular compromise for attached probes were higher in terms of blood flow and hemoglobin oxygen saturation in RFFF than in ALTF [7, 9]. The differences with respect to a cut-off value of an increase in hemoglobin concentration greater than 10% for attached surface probes and greater than 30% for unattached surface probes may be related to the tendency for generally higher hemoglobin concentration values with attached surface probes, measuring at a tissue depth of 3 mm, compared with unattached surface probes, measuring at tissue depths of 2 mm and 8 mm [7, 9].

This study found that flap perfusion monitoring with attached surface probes for the O2C analysis system on the basis of all flap perfusion parameters combined — i.e., blood flow, hemoglobin concentration, and hemoglobin oxygen saturation — yielded a sensitivity of 100.0%, a specificity of 97.1%, and an accuracy of 97.1% in detecting vascular compromise, relative to flap perfusion monitoring with attached surface probes for the O2C analysis system serving as the reference standard method. Interestingly, some measurements with attached surface probes showed hemoglobin oxygen saturation values indicative of vascular compromise, whereas this was not the case with unattached surface probes. This resulted in a specificity of 97.1% for hemoglobin oxygen saturation evaluated separately but also possibly reflects the potential of flap perfusion monitoring with attached surface probes in the earlier detection of vascular compromise. In addition, based on a Cohen’s kappa of 0.653, the study found substantial concordance between flap perfusion monitoring with attached and unattached surface probes [18]. However, given the low specificity value, the concordance between attached and unattached surface probes for hemoglobin oxygen saturation evaluated separately was only fair, with a Cohen’s kappa of 0.325 [18].

In general, the study demonstrated that flap perfusion monitoring with attached surface probes for the O2C analysis system was comparable to flap perfusion monitoring with unattached surface probes in terms of technical feasibility and patient safety, as no event of probe detachment or disconnection and no infections or bleeding due to the attachment structures were observed as potential disadvantages of the attached surface probes [26].

Nevertheless, the study has several limitations, such as the small number of patients in the sample, the limited number of ALTFs included in the study, and the fact that only four flaps in the study had vascular compromise and subsequently underwent flap revision. With regard to the limited number of four flaps that required revision, it should be noted that in microvascular reconstruction of the head and neck region, the number of free flaps that require revision is generally low; the percentage of flaps that required revision in this study, 13.3%, was within the range observed in the literature [8, 27–29]. However, the determination of cut-off values indicative of vascular compromise and the assessment of values such as sensitivity, specificity, and accuracy in terms of the detection of vascular compromise for attached surface probes can only be considered as approximative values and a first orientation in this context. Furthermore, all revised RFFFs showed primarily venous vascular compromise and therefore likely only secondarily altered values for blood flow; in addition, the only revised ALTF showed primarily arterial vascular compromise and therefore likely only secondarily altered values for hemoglobin concentration [7, 9]. Regarding the limited postoperative monitoring period in this study up to 48 h postoperatively, and the potential occurrence of the requirement for flap revision beyond this period, it should be kept in mind that the risk of vascular compromise in free flaps is highest during the first period of 48 h postoperatively, and the flap salvage rate is lower beyond the period of 48 h postoperatively [11, 30, 31].

This study evaluated for the first time postoperative flap perfusion monitoring with attached surface probes for the O2C analysis system in terms of detecting vascular compromise in microvascular head and neck reconstruction. The study demonstrated that attached surface probes are technically feasible and represent a safe option for patients to be used with the O2C analysis system for postoperative flap perfusion monitoring. Based on distinctive cut-off values, flap perfusion monitoring with attached surface probes showed high accuracy and, more importantly, a substantial concordance with flap perfusion monitoring with unattached surface probes, supporting interchangeability between the two surface probes. Further studies are needed to confirm the cut-off values indicative of vascular compromise for attached surface probes and to validate their accuracy to maintain the clinical utility of the O2C analysis system for flap perfusion monitoring.

## Conclusion

This study demonstrated that flap perfusion monitoring with attached surface probes for the O2C analysis system was comparable to the standard use with unattached surface probes in terms of technical feasibility and patient safety. Based on cut-off values indicative of vascular compromise (blood flow: RFFF < 60AU and ALTF < 40AU; hemoglobin concentration: RFFF > 100AU or > 10% increase and ALTF > 80AU or > 10% increase; hemoglobin oxygen saturation: RFFF < 40% and ALTF < 30%), flap perfusion monitoring with attached surface probes showed an accuracy of 97.1% in detecting vascular compromise and may form the basis for continuous flap perfusion monitoring with the O2C analysis system.

## Data Availability

The data underlying this article is available on reasonable request to the corresponding author.
